# Supplementary material for: The ATM-E6AP-MASTL axis mediates DNA damage checkpoint recovery
Source: bioRxiv. 2023 Feb 22:2023.02.22.529521. Preprint. [Version 1] doi: 10.1101/2023.02.22.529521 (PMC9980089; doi:10.1101/2023.02.22.529521)

bioRxiv preprint doi: https://doi.org/10.1101/2023.02.22.529521; this version posted February 22, 2023. The copyright holder for this preprint (which was not certified by peer review) is the author/funder, who has granted bioRxiv a license to display the preprint in perpetuity. It is made available under aCC-BY 4.0 International license.

The ATM-E6AP-MASTL axis mediates DNA damage checkpoint recovery

Yanqiu Li[1], Feifei Wang[1], Xin Li[1], Ling Wang[1], Zheng Yang[2], Zhongsheng You[2], and Aimin Peng[1,*]

1. Department of Oral Biology, University of Nebraska Medical Center, Lincoln, Nebraska, USA

2. Department of Cell Biology and Physiology, School of Medicine, Washington University in St. Louis, St. Louis, Missouri, USA

* Author for correspondence: aimin.peng@unmc.edu

Key words: DNA damage checkpoint, cell cycle recovery, MASTL, E6AP, ATM

Figure 1 Supplemental 1. MASTL upregulation after DNA damage.

(A, B) SCC38 cells were treated with 10 mM HU (A) or 10 Gy IR (B), and incubated for 0-10 hours, as indicated. The expression levels of MASTL, RPA, and α-tubulin were analyzed by immunoblotting. (C, D) HeLa cells were treated with 0.5 uM DOX (C) or 10 mM HU (D) as indicated, cell lysates were collected and analyzed by immunoblotting for MASTL and α-tubulin.

Figure 1 Supplemental 2. The expression levels of cell cycle kinases after DNA damage.

HeLa (A) or SCC38 (B) cells were treated without or with 2 mM of hydroxyurea (HU) for 8 hours (A), or 3-6 hours (B). Cell lysates were harvested for immunoblotting.

bioRxiv preprint doi: https://doi.org/10.1101/2023.02.22.529521; this version posted February 22, 2023. The copyright holder for this preprint (which was not certified by peer review) is the author/funder, who has granted bioRxiv a license to display the preprint in perpetuity. It is made available under aCC-BY 4.0 International license.

Figure 2 Supplemental 1. Increased protein stability of MASTL after DNA damage. HEK293 cells were treated without (A) or with (B) 10 mM HU for 2 hours. These cells were then treated with cycloheximide (CHX, 20 μg/ml) at time 0 to block protein synthesis, and analyzed by immunoblotting for the protein stability of MASTL and α-tubulin.

Figure 3 Supplemental 1. E6AP and MASTL associate via their N-terminal motifs. (A) MBP-E6AP and GST-MASTL were expressed and purified, as described in Materials and Methods. GST-MASTL on glutathione beads, or control glutathione beads, were incubated with MBP-E6AP, followed by pulldown, as described in Materials and Methods. The input, GST-MASTL pulldown, and control pull down samples were analyzed by immunoblotting for MASTL and E6AP. (B) GFP-MASTL, FL or N-terminus (aa 1-340), was expressed in HeLa cells. Immunoprecipitation (IP) was performed using a GFP antibody, and the presence of E6AP in the IP products was examined by immunoblotting. (C) IP was performed using GFP-tagged segments of E6AP expressed in HeLa cells. The IP product, cell lysate input, and a control IP (using empty beads) were analyzed by immunoblotting for MASTL and GFP. N: aa 1-280; N1: aa 1-99; N2: aa 100-207; N3: aa 108-280.

Figure 4 Supplemental 1. E6AP mediates MASTL ubiquitination and degradation. (A) HeLa cells were treated without or with E6AP siRNA. The RNA levels of E6AP and MASTL were quantified by real-time PCR. The mean values were calculated from three experiments. Statistical significance was determined using an unpaired 2-tailed Student's

bioRxiv preprint doi: https://doi.org/10.1101/2023.02.22.529521; this version posted February 22, 2023. The copyright holder for this preprint (which was not certified by peer review) is the author/funder, who has granted bioRxiv a license to display the preprint in perpetuity. It is made available under aCC-BY 4.0 International license.

t test. A p-value more than 0.05 was considered non-significant (ns). (B) The E6AP *in vitro* ubiquitination assay was performed using MASTL as substrates, as in Fig. 4H. S5a was added as a control substrate. E1/2 and E3 (E6AP) enzymes were added in the reactions, as indicated. Samples were analyzed by immunoblotting for MASTL and S5a after 90 min incubation. (C) *In vitro* ubiquitination assay was performed using E6AP as E3 ligase, and ΔN MASTL (aa 335-887) as substrate. S5a was added as a control substrate. The reactions were incubated as indicated, as analyzed by immunoblotting for MASTL and S5a.

Figure 5 Supplemental 1. Impaired DNA damage checkpoint signaling in E6AP-null cells.

WT or E6AP knockout (KO) HeLa cells were treated with 0.1 μM etoposide (ETO) for the indicated hours. Cells were analyzed by immunoblotting for phospho-ATM/ATR substrates and α-tubulin.

Figure 7 Supplemental 1. DNA damage-induced E6AP Ser-218 phosphorylation is mediated by ATM.

(A) WT or E6AP knockout HEK293 cells were treated without or with doxorubicin (DOX, 0.5 μM) for 4 hours. Cells were harvested and analyzed by immunoblotting for phospho-E6AP Ser-218 and α-tubulin. (B) HEK293 cells were treated without or with DOX (0.5 μM), or ATM inhibitor (KU55933, 10 μM), and analyzed by immunoblotting. (C) HeLa cells were treated with hydroxyurea (HU, 10 mM) combined with ATM/ATR

bioRxiv preprint doi: https://doi.org/10.1101/2023.02.22.529521; this version posted February 22, 2023. The copyright holder for this preprint (which was not certified by peer review) is the author/funder, who has granted bioRxiv a license to display the preprint in perpetuity. It is made available under aCC-BY 4.0 International license.

inhibitor (caffeine, 4 mM) or ATM inhibitor (KU55933, 10 μM), for 12 hours, as indicated. Cells were harvested and analyzed by immunoblotting.

Figure 1 Supplemental 1

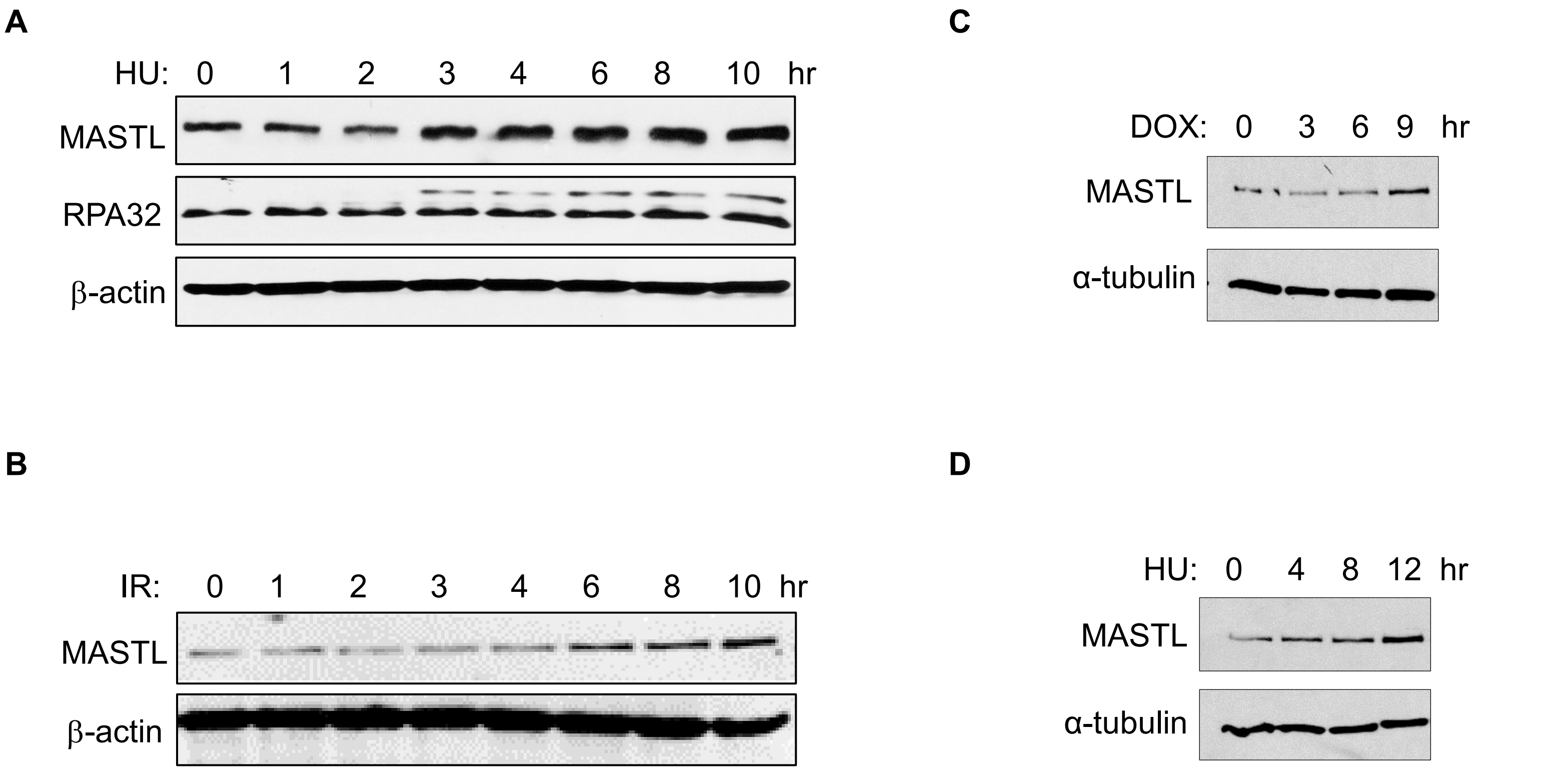

Figure 1 Supplemental 2

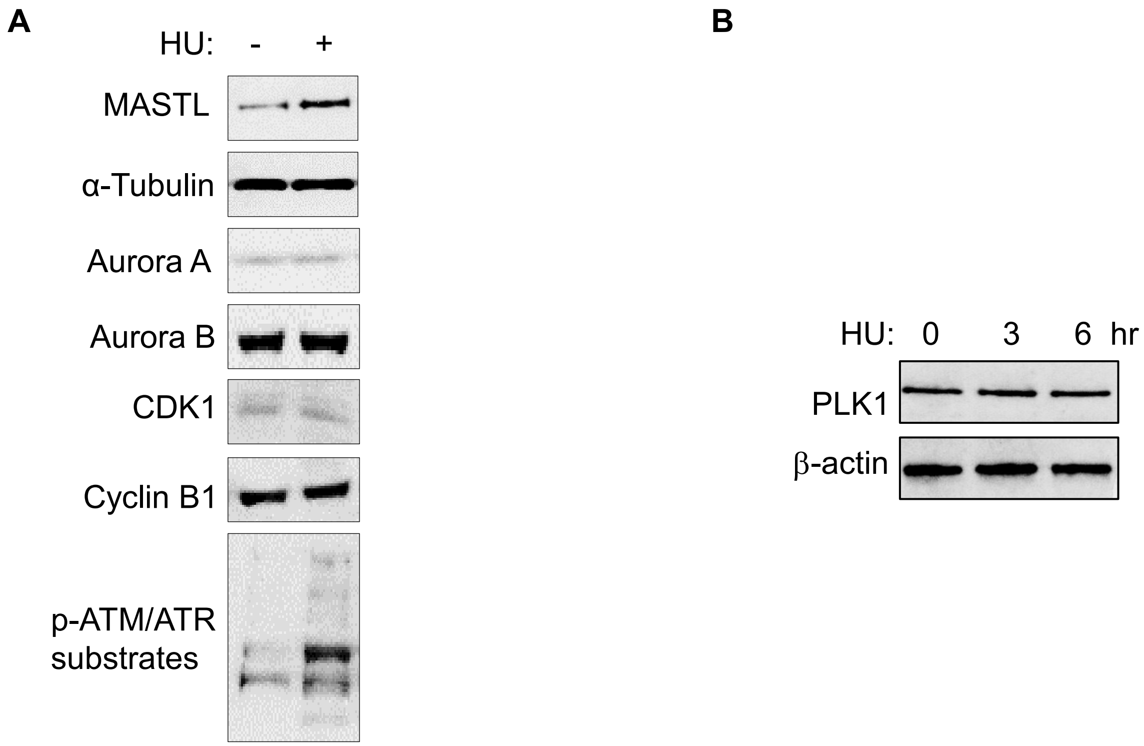

Figure 2 Supplemental 1

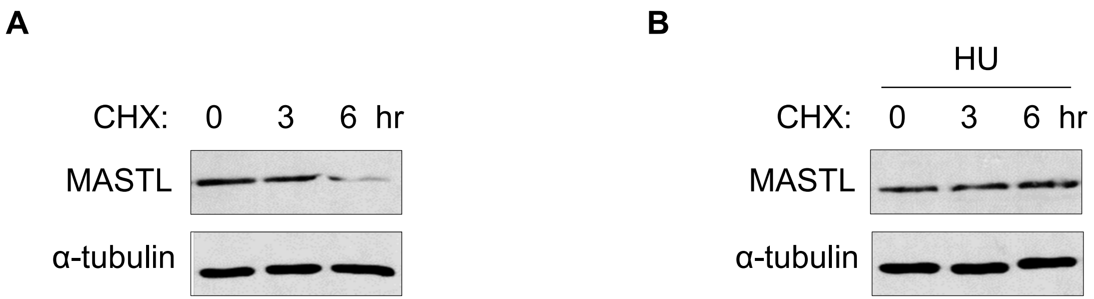

Figure 3 Supplemental 1

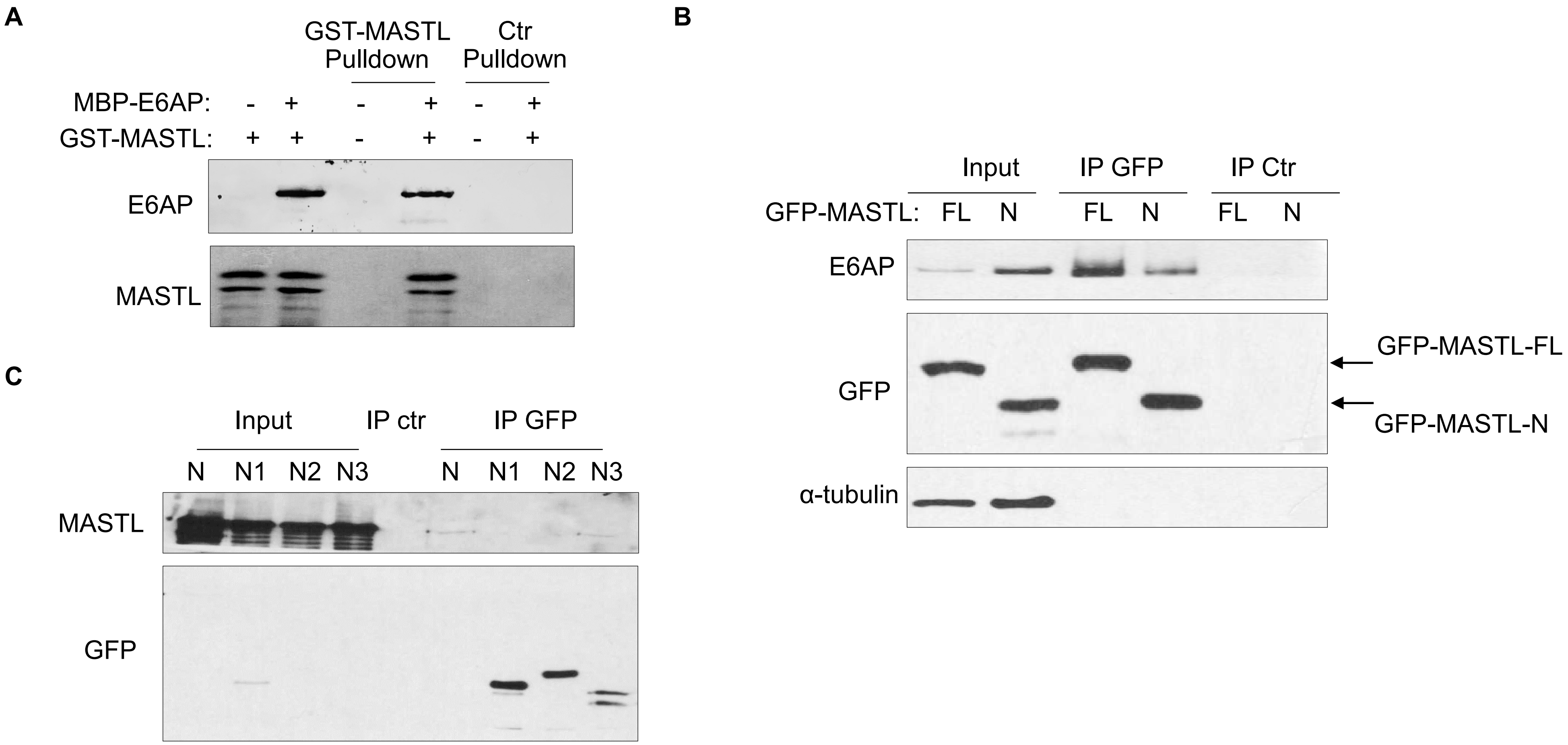

Figure 4 Supplemental 1

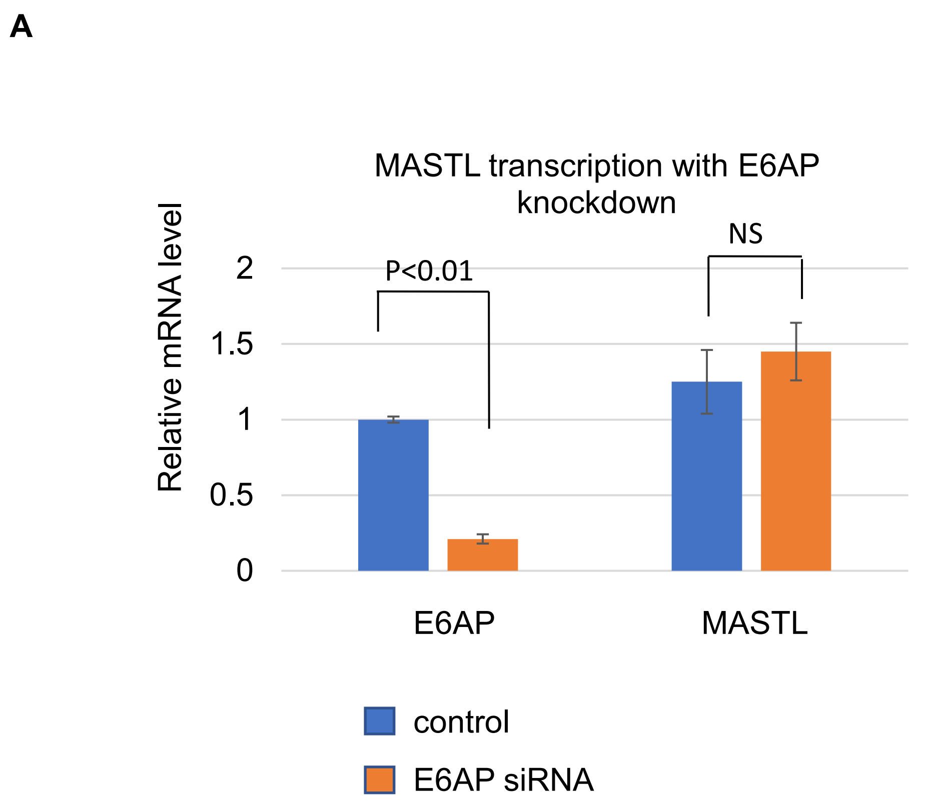

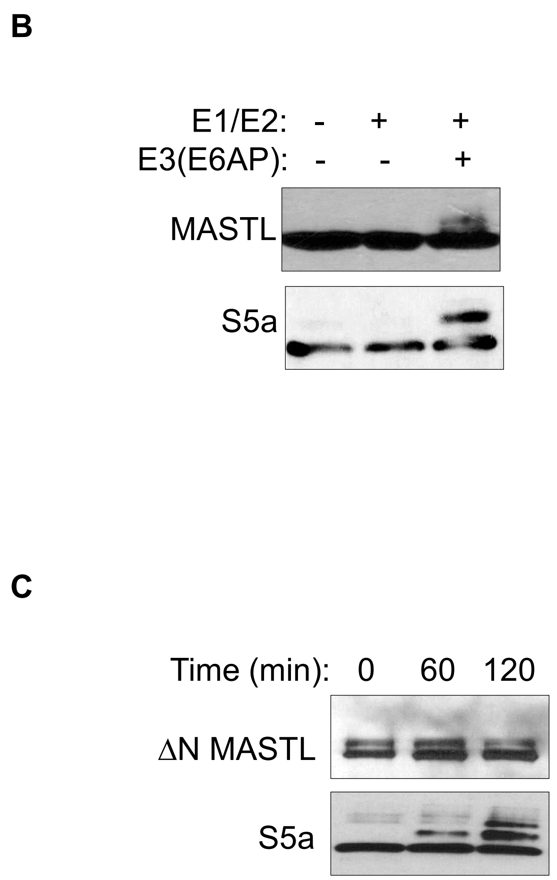

Figure 5 Supplemental 1

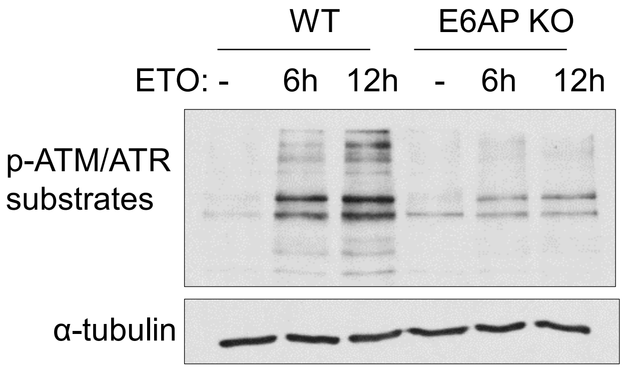

Figure 7 Supplemental 1

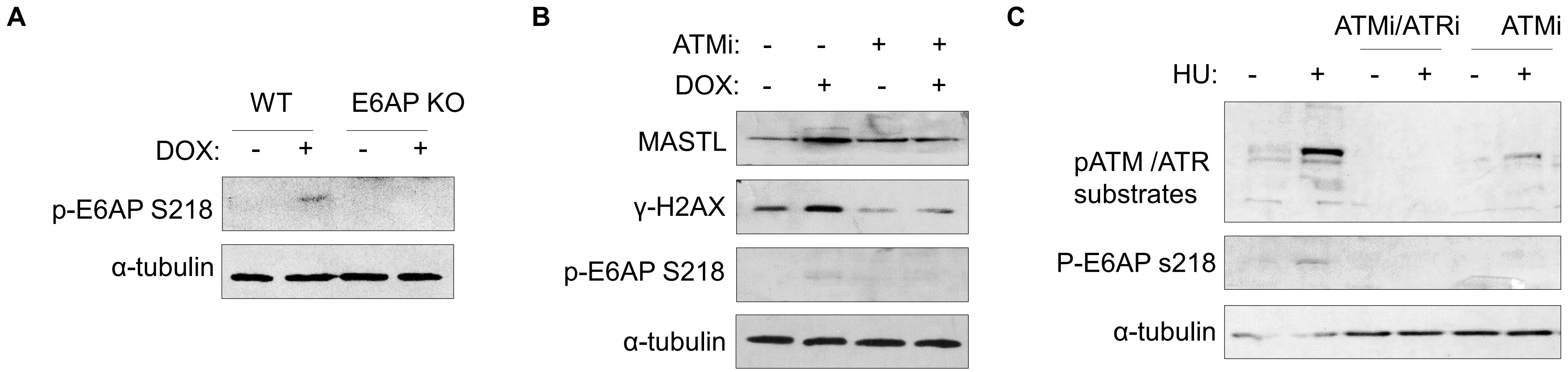

Supplement: Supplementary file 1 [file NIHPP2023.02.22.529521v1-supplement-1.pdf]
